# A “cell-free treatment” for tendon injuries: adipose stem cell-derived exosomes

**DOI:** 10.1186/s40001-022-00707-x

**Published:** 2022-05-28

**Authors:** Kexin Lyu, Tianzhu Liu, Yixuan Chen, Jingwei Lu, Li Jiang, Xueli Liu, Xinyue Liu, Yujie Li, Sen Li

**Affiliations:** 1 School of Physical Education, Southwest Medical University, Luzhou, China; 2 Neurology Department, The Affiliated Traditional Chinese Medicine Hospital of Southwest Medical University, Luzhou, China; 3 Spinal Surgery Department, The Affiliated Traditional Chinese Medicine Hospital of Southwest Medical University, Luzhou, China

**Keywords:** Exosomes, Adipose-derived stem cells, Tendon, Mechanism, Role

## Abstract

Tendon injuries are widespread and chronic disorders of the musculoskeletal system, frequently caused by overload of the tendons. Currently, the most common treatment for tendon injuries is "cell-free therapy", of which exosomes, which can treat a host of diseases, including immune disorders, musculoskeletal injuries and cardiovascular diseases, are one kind. Among the many sources of exosomes, adipose-derived stem cell exosomes (ASC-Exos) have better efficacy. This is attributed not only to the ease of isolation of adipose tissue, but also to the high differentiation capacity of ASCs, their greater paracrine function, and immunomodulatory capacity compared to other exosomes. ASC-Exos promote tendon repair by four mechanisms: promoting angiogenesis under hypoxic conditions, reducing the inflammatory response, promoting tendon cell migration and proliferation, and accelerating collagen synthesis, thus accelerating tendon healing. This review focuses on describing studies of preclinical experiments with various exosomes, the characteristics of ASC-Exos and their mechanisms of action in tendon healing, as well as elaborating the limitations of ASC-Exos in clinical applications.

## Introduction

Tendons, which connect muscle to the bone, are deep-set structures of the body [1, 2]. Normal tendons are bright white, consisting of numerous collagen fibers arranged in parallel [3]. Tendon injuries are the most common diseases in the musculoskeletal system, accounting for 30–40%, and are generally accompanied by a vast array of pathological phenomena, including tendon swelling, local pain, and functional impairment [4, 5]. In most cases, overuse and overloading of tendons can contribute to injuries [6], while other factors such as age, metabolism, blood pressure can also aggravate tendon injuries [7, 8]. Once the pain exacerbates, surgical treatment will be needed [9]. Tendon injuries place a mental and physical burden on all susceptible individuals [10]. Therefore, further experiments are needed to verify the efficacy of a wide range of methods on tendon injuries [11].

**Fig. 1:**
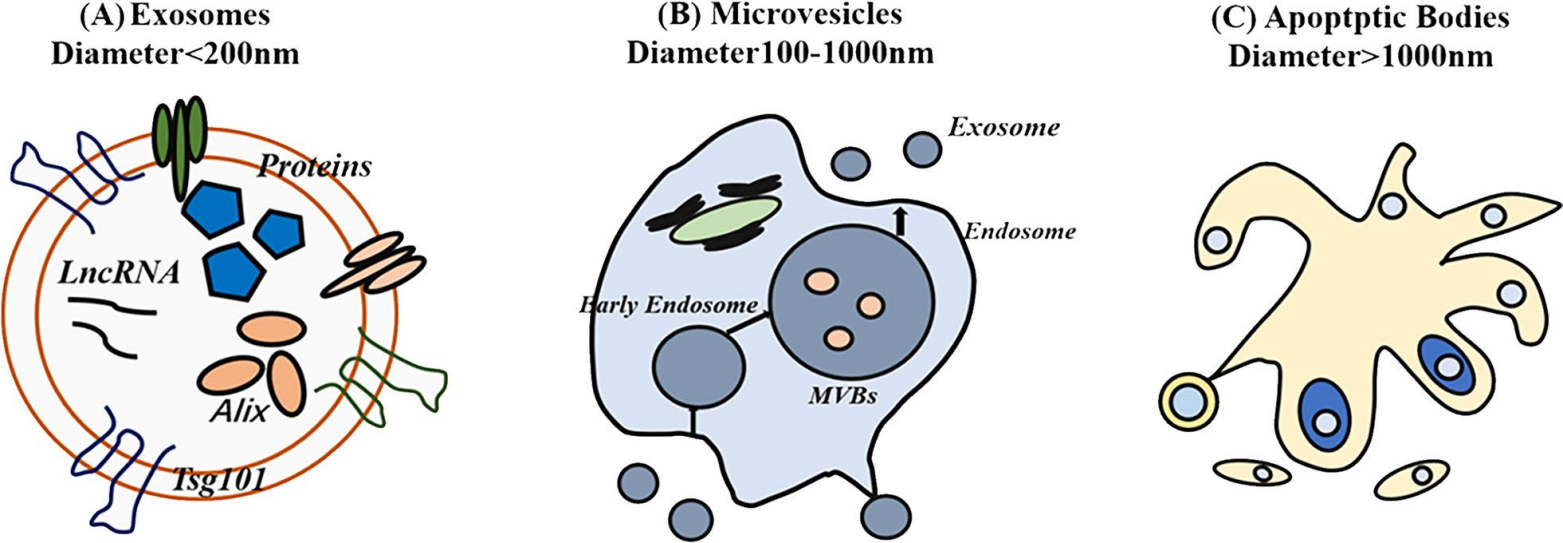
The classification of extracellular vesicles

It has been documented that therapeutic modalities for tendon injuries can be divided into two types: surgical and non-surgical methods [12, 13]. In surgical methods, the treatments for tendon injuries universally rely on operative procedures and non-steroidal anti-inflammatory drugs [14, 15]. In terms of non-surgical approaches, currently, cell-free therapy in regenerative medicine ranks among the top treatments for tendon injuries, with the most prominent source being the secretome of stem cell origin [16]. The benefits of this therapy are manifold, including a reduced immunogenic response, maintenance of post-storage activity, the ability to target characteristic tissues, and a higher safety profile [17–19]. Based on several in vivo and in vitro experiments, stem cell-derived exosomes can treat quite a few diseases, such as myocardial infarction, burns, and pulmonary fibrosis, with superior results compared to using stem cells exclusively [17, 20, 21]. Among the numerous types of cell-free therapies, exosomes from adipose stem cells (ASC-Exos) are one of the most promising ways to promote tendon healing [18]. Exosomes are extracellular vesicles with nanoscale bilayer membranes, and are secreted by diverse types of stem cells [22]. The classification of extracellular vesicles is shown in Fig. 1.

The majority of investigations have demonstrated that ASC-Exos contain a broad range of proteins of intracellular origin, which are engaged in intracellular signaling, diverse catalysis, etc. [23]. Moreover, these exosomes act on more than two hundred signaling pathways, including phosphatidylinositol-3-kinase/protein kinase B (PI3K/Akt), Jak-STAT and wingless-associated integration site (Wnt) pathways, which can subsequently be involved in cell differentiation and proliferation, thus enhancing tissue regeneration [24, 25]. It has been suggested that ASC-Exos play a pivotal role in a wide range of physiological or pathological processes in tendons, especially in the immune response, apoptosis, and tendon repair, which are of direct relevance to the mechanical properties of tendons [26–28]. Additionally, there are already manifold studies expressing their apparent effect in the treatment of Alzheimer's disease, because ASC-Exos can markedly raise the survival rate of human neuroblastoma cells, and in some long-term diseases we can use ASC-Exos as an emerging treatment modality [29, 30]. Moreover, the primary mechanisms of ASC-Exos for tendon repair involve the following aspects: the promotion of angiogenesis, the proliferation and migration of tenocytes, the production of anti-inflammatory factors, the maintenance of metabolic homeostasis, and the synthesis of collagen [19, 28, 31, 32].

Although there are numerous in vitro trials that have been able to prove the substantial effects of exosomes on tendon repair [33], further in vivo studies using direct methods are needed to determine the roles played by the ASC-Exos. In addition, the usage of exosomes for tendon injuries recontinues to be restricted in some ways. Since the process of exosome use involves a growing body of signaling pathways, alterations of several biological factors and cytokines, there remain ambiguities in the present study of its mechanisms. Hence, after taking into account the role played by exosomes in the process of tendon regeneration, their future therapeutic application needs to be refined in terms of dose and duration of use [24]. Although exosomes as a cell-free therapy offer a promising therapeutic modality, we need to further subdivide them at the proteomic, lipidomic or nucleic acid level in order to refine them as targeted therapeutic modalities [33]. This review aims to summarize the roles of ASC-Exos in the treatment of tendon injuries and to give an overview of existing evidence, which could assist in responding to these important questions.

## Methods

**Fig. 2:**
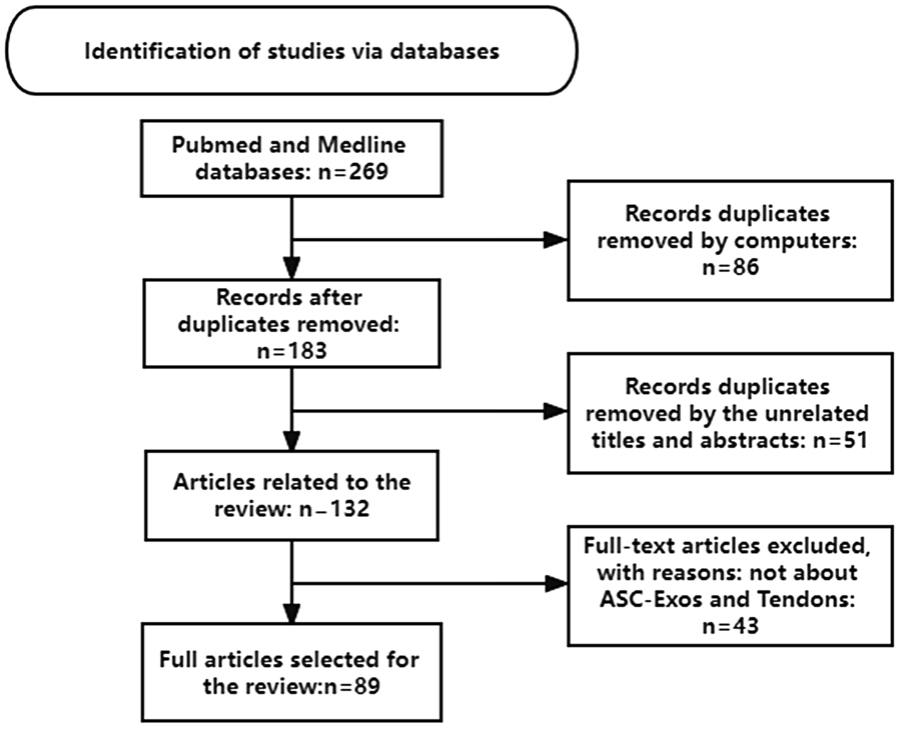
Article retrieval flowchart with inclusion and exclusion process

(i) Search site: articles are from PubMed, a database of papers on biomedical science. (ii) Database: MEDLINE. (iii) Keywords: Exosomes; Adipose-derived stem cells; Tendon; Mechanism. (iv) Boolean algorithm: ((((Extracellular vesicles) OR (exosomes)) AND (stem cells)) OR (Adipose-derived stem cells)) AND (tendon) (v) Retrieving timeframe: we searched the selected journals published from 2008 to 2021. (vi) Inclusion and exclusion criteria: articles were included if the topic is related to exosomes and tendon repair, while the article type was a review or an experimental paper. The retrieval process is shown in Fig. 2.

## Illustrations of different sources of exosomes in preclinical studies

The secreted tissues can be classified as tendon stem cells (TSCs), macrophages, human umbilical cord mesenchymal stem cells (HUMSCs), bone marrow mesenchymal stem cells(BMSCs), adipose-derived stem cells (ASCs), etc., depending on the source of the exosomes [19, 33–35]. Several trials have documented the role of exosomes in maintaining tendon homeostasis, which includes cell proliferation and differentiation, extracellular matrix changes and collagen synthesis, and other changes that affect the mechanical properties of tendon tissue [28]. While numerous basic experimental studies have been conducted on these exosomes, there is a paucity of research on their clinical application. The relevant effects of various exosomes on tendon injury are shown in s Table 1. The main biomolecules and signaling pathways involved in the roles of different exosomes in the treatment of tendon injuries are shown in Table 2.

**Table 1:** Therapeutic effects of exosomes from three sources in tendon injury

ASC-Exos	BMSC-Exos	TSC-Exos
Compared with exosomes from other sources, ASC-Exos gradually become the main substances used in "cell-free therapy". ASC-Exos modulate the phenotype of macrophages, suppress the inflammatory response, improve the immune effect, stimulate the proliferation and migration of tendon cells, and maintain tendon metabolic homeostasis, thus improving the expression of histological and mechanical properties of damaged tendons. Moreover, some pretreatments (e.g., hypoxia or chemical treatment) can further modulate the immunosuppressive effects	BMSC-Exos have been very well received in clinical practice as the first "cell-free therapy" technology to be implemented. They promote angiogenesis, inhibit the secretion of pro-inflammatory factors to slow down the inflammatory response, as well as modulate some immune system related biological factors and encourage the regenerative capacity of tendon resident stem/progenitor cells. Therefore, BMSC-Exos can be one of the effective therapeutic tools for the treatment of immune-related diseases	TSC-Exos mainly act on the proliferation and migration of tenocytes, thereby indirectly promoting tendon healing. They mainly change the ratio of matrix metalloproteinase 3 and its inhibitors, regulate the degradation and synthesis of ECM, and enhance the mechanical properties of tendons

**Table 2:** The main biomolecules and signaling pathways involved in the roles of different exosomes in the treatment of tendon injuries

Exosomes	Angiogenesis	Reduce inflammatory response	Changes in tendon cells	Regulation of immune response	Fibrosis
ASC-Exos	(1) HIF-α**↑** (2) VEGF**↑** (3) miRNA-125a**↑** (4) MMP-2**↑**	(1) AMPK**↑** (2) Wnt/β**↓** (3) IL-1β**↓** (4) IL-6**↑**	(1) MEK/ERK1/2**↑** (2) PI3K/Akt**↑** (3) The SMAD2/3 and SMAD1/5/9 signaling pathway**↑**	(1) M2**↑** (2) Wnt/β-catenin**↑** (3) notch signaling pathway**↑** (4) miR-21**↑**	(1) MMP-9**↓** (2) MMP-13**↓**
BMSC-Exos	(1) VEGF**↑** (2) Hippo**↑**	(01) Anti-inflammatory factors**↑** (2) Pro-inflammatory factors**↓** (3) Macrophages polarization**↑**	(1) MEK/ERK1/2**↑** (2) PI3K/Akt**↑**	(1) Pro-inflammatory factors**↓** (2) Treg differentiation (3) M2 polarization	TGF-β**↓**
TSC-Exos	BMP12**↑**	/	(1) MEK/ERK1/2**↑** (2) PI3K/Akt**↑** (3) BMP/Smad?	/	the MIR-15b-5p/FGF-1/7/9 pathway↑
UC-MSC-Exos	HIF-1α/VEGF↑	**/**	(1) PTEN/ mTOR/TGF-β1↑ (2) miR-29a-3p↑	**/**	(1) miR-21a-3p**↓** (2) p-p65,COX2**↓**
Exosomes from macrophages	Macrophage-derived exosomes act on tendon injury mainly by promoting macrophage polarization, followed by the production of different cytokines by M1 and M2 macrophages, respectively, thus promoting tendon repair				

TSC-Exos play an influential role in enhancing tendon injury healing. In vitro experiments, TSC-Exos have been shown to mainly affect the proliferation and migration of tendon cells, while in extensive in vivo experiments, TSC-Exos have demonstrated a major impact on aspects of inflammatory response and tendon regeneration after tendon injury [36]. In particular, TSC-Exos can alter the tendon healing ability by regulating the synthesis and degradation of extracellular matrix, as well as modifying the mechanical properties of the tendons [37].

In turn, macrophage-derived exosomes promote fibrosis after tendon injury mainly through the MIR-15b-5p/FGF-1/7/9 pathway in the context of mediating circRNA-Ep400, which not only reveals the mechanism of association between macrophages and stromal cells, but also explores the role of exosomes in the process of tendon adhesion. However, further experiments are still required to verify their effectiveness on scar adhesion [38, 39].

In the late twentieth century, BMSCs were initially discovered and introduced, and consequently, for a lengthy period, bone marrow served as an essential source of the exosomes involved in the use of “Stem Cell-Free Therapy” [40]. It has been demonstrated that exosomes released from BMSCs can transmit pertinent biological signals and subsequently perform at the impaired site, and these BMSC-Exos have multiple merits compared to injecting only BMSCs into the lesion area [22, 35]. BMSC-Exos primarily enhance angiogenesis by boosting the increases in vascular endothelial growth factor (VEGF) and activating the Hippo signaling pathway, while they inhibit the polarization of macrophages and accelerate the secretion of anti-inflammatory factors by M2 macrophages, thus reducing inflammation and ultimately facilitating tendon repair [41, 42].

ASC-Exos are attracting more and more attention compared to the aforementioned exosomal sources, primarily owing to two benefits of adipose tissue: firstly, ASCs can be obtained relatively easily from adipose tissue, and secondly, ASCs are widely spread in the body and have a high proliferation rate [43]. In this regard, a considerable volume of experiments conducted on the rotator cuff and the Achilles tendon in mice have clearly identified the abilities of these exosomes to accelerate tendon healing [19, 27].

ASC-Exos work effectively in certain musculoskeletal disorders (osteoarthritis, tendinopathy) [44]. Since ASC-Exos work in a dose-dependent manner, their clinical use can alter following the diseases [45]. For example, depending on the wound-containing models of the organism, in vitro experiments have shown that the optimal concentration for ASC-Exos use remains 50 μg/ml, while for lipogenic differentiation the concentration is approximately 40 μg/ml [45]. The appropriate dosage level for the treatment of tendon injuries lies at 100 μg/ml, a concentration that rapidly activates certain signaling pathways and consequently stimulates the proliferation of tendon cells [19]. Further investigations still need to be pursued to verify the optimal dose and the frequency of injection.

## The roles and mechanisms of ASC-Exos in tendon healing

**Fig. 3:**
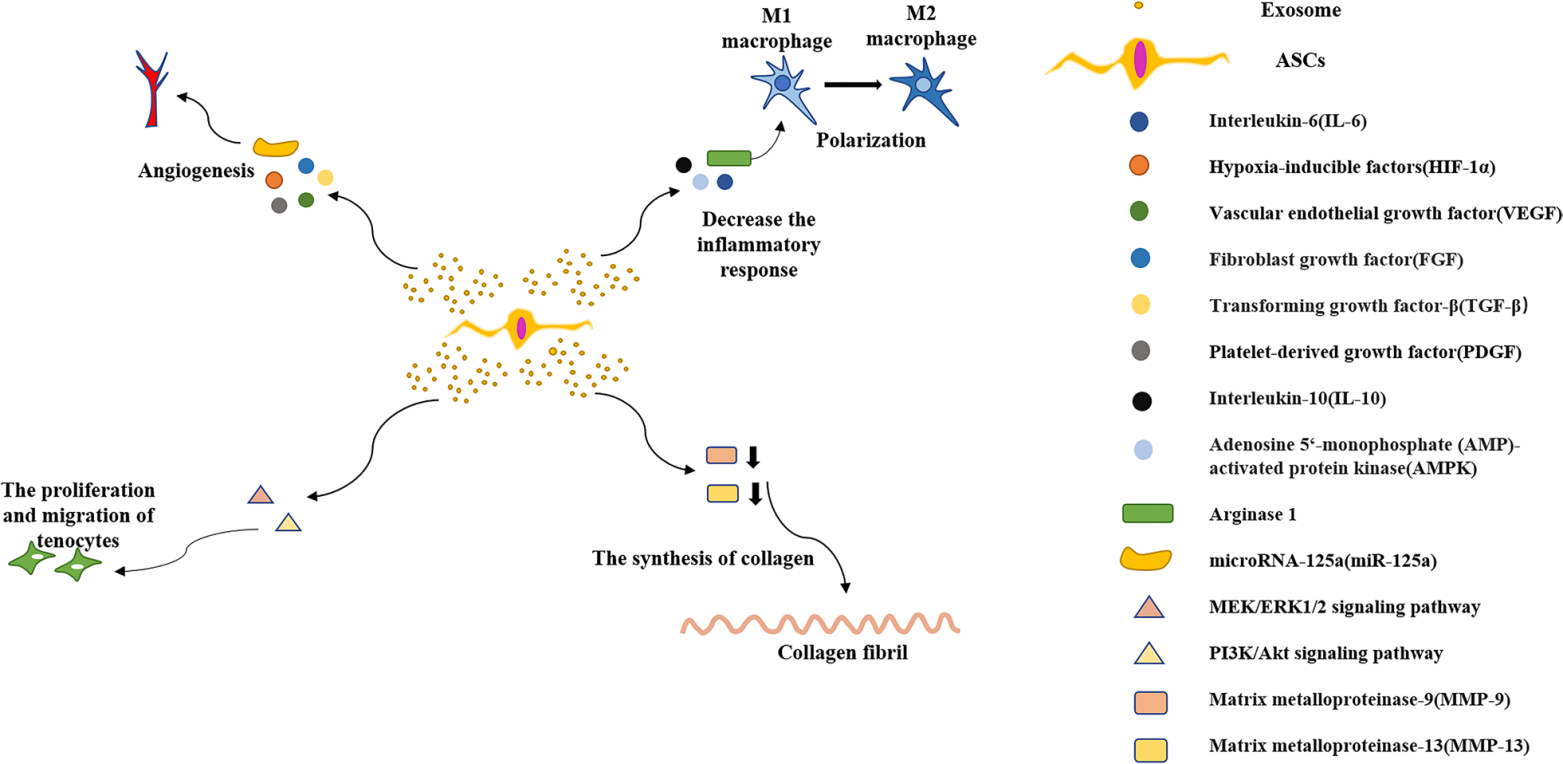
The roles and mechanisms of ASC-Exos in the treatment of tendon injury

Exosomes are composed of three main substances: lipids, proteins, and RNA [22]. In particular, exosomes contain a substantial set of microRNAs that regulate intercellular communication processes and serve in the inflammatory response, fibrosis and tendon repair [46]. Accordingly, exosomes are engaged in a multitude of biological reactions in the organism, and they transmit messages to a wide range of systems by modulating bio-factors and activating signaling pathways [47]. Some specific substances contained in exosomes are shown in Table 2. At this point, the markers associated with ASC-Exos that can be observed microscopically are tetralipoprotein, CD81 and CD63 [48, 49]. Furthermore, in certain cases exosomes have been shown to have full therapeutic effect of stem cells in specific disorders, notably in retinal injury, myocardial ischemia–reperfusion injury, and pulmonary hypertension [50]. Collectively, a vast array of investigations have illustrated that in addition to being vital for inter-biomolecular linkages, exosomes are capable of well regulating quite a few metabolic reactions and relatively unique bio-processes of the body, ranging from anti-inflammation, enhancing angiogenesis, reducing apoptosis, and participating in immunomodulatory responses [40]. The roles and mechanisms of ASC-Exos in tendon healing are shown in Fig. 3.

### ASC-Exos promote angiogenesis

During the first stage of tendon repair—the inflammatory phase. This stage is typically accompanied by a substantial rise in growth factors, cytokines, and angiogenic factors, which trigger a host of bio-reactions and thereby lead to alterations in vascular permeability, thus indirectly supporting angiogenesis [51, 52]. It is now well established that ADSC-Exos, by stimulating angiogenesis, will raise the probability of nerve regeneration in fat grafts, thereby modulating the inflammatory response and ultimately expediting the rate of tendon healing [19, 27, 45].

In principle, the capacity of ADSC-Exos to drive angiogenesis under hypoxic conditions will rise remarkably, and it can significantly elevate the level of hypoxia-inducible factors (HIF-1α) [53]. This is due to the fact that HIF-1α, a factor involved in mediating the expression of diverse genes while enhancing the ability of cells to survive in a hypoxic environment, can trigger the pathway of angiogenesis [54]. It has been proposed that in a hypoxic environment tendon cells and ASC regulate intercellular signaling and extracellular matrix communication through the contents of exosomes, which triggers their paracrine action and thus maintains tendon homeostasis [55]. ADSC-Exos are packed with growth factors (fibroblast growth factor, glial cell line-derived neurotrophic factor) and angiogenic factors (hepatocyte growth factor, hairy and enhancer of split 1, vascular endothelial growth factor) [31], among which VEGF is a signaling protein most closely linked to angiogenesis. Its actions chiefly include promoting the proliferation of endothelial cells, enhancing vascular permeability, strengthening the migration of cells, and improving neovascular adaptation [45, 56]. ADSC-Exos consist of multiple species of miRNAs, which include miR-125a, miR-126, and miR-132, in which miR-125a has a major impact on angiogenesis [57]. It has been partially demonstrated that two types of miRNA secreted by ASC-Exos, miR-23a and miR-23b, can both promote angiogenesis, but these two miRNAs failed to detect in the tendinopathy model; therefore, miRNA could be a novel aspect for detection in future studies [58]. The role of ADSC-Exos in the initial process of tendon healing is to primarily trigger the secretion of matrix metalloproteinase (MMP-2) and inhibit the transfer of miR-125a from endothelial cells to other tissues while targeting specific regions to reduce the number of angiogenesis inhibitors, followed by upregulation of VEGF and FGF together with an increase in the migration rate of vascular endothelial cells, thereby promoting angiogenesis and ultimately accelerating the rate of tendon healing [45, 57].

In addition to the above angiogenesis-related biological factors, the impact of platelet-derived growth factors (PDGF) secreted by adipose-derived stem cells in the process of angiogenesis should not be neglected [40]. However, there remains evidence in the literature that ADSC-Exos does not boost angiogenesis, but rather suppresses its effects, probably owing to the dual effect of ADSC-Exos. In experiments, ADSC-Exos downregulate VEGF, which is achieved in a concentration-dependent manner [59]. Further experiments are necessary to verify the effect of ADSC-Exos on angiogenesis in specific musculoskeletal system disorders.

### ASC-Exos decrease the inflammatory response

Following tendon injury, its intracellular metabolic balance is disrupted, and these alterations in it consist of changes in inflammatory response-associated factors and expression of matrix metalloproteinases, which leads to degenerative modifications of the tendon and thus prolongs its healing time [28]. There are cases where ADSC-Exos have been shown to significantly modulate the inflammatory response, including reducing the infiltration of inflammatory cells and enhancing the release of anti-inflammatory factors and modulating the associated immune response and accelerating the healing ability of tendons [35, 45]. The biological factors related to inflammatory response are shown in Table 3.

**Table 3:** Diverse pro-inflammatory substances in the inflammation phase

Diverse pro-inflammatory substances	Functions	References
TNF-α	Tumor necrosis factor α, is a pro-inflammatory cytokine, which affects other factors in tendon repair	[60]
IL-1β	Interleukin-1, is produced by the macrophages, monocytes and dendritic cells, which inhibits the formation of cartilage matrix	[61, 62]
IL-6	Interleukin-6 is a pro-inflammatory factor that plays a central role in tissue injury	[61]
COX-2	Cyclooxygenase-2, a key enzyme for conversion of arachidonic acid to prostaglandins	[63]

Collectively, maintaining metabolic homeostasis after a tendon injury is achieved by enhancing the signaling of adenosine 5ʹ-monophosphate (AMP)-activated protein kinase (AMPK) while inhibiting the activity of Wnt/β-catenin [28]. Initially, ADSC-Exos promote the signaling of AMPK, a regulator that can mediate the gene expression of biomolecules and thus influence a wide range of physiological activities [28]. This is followed by a decrease in Wnt/β-catenin activity, one of the pathways involved in cytogenesis, where β-catenin is an effector of Wnt signaling, a protein that accelerates the inflammatory response and slows down the cellular metabolic process [64]. Additionally, the activation of Wnt-β–catenin pathway is closely related to MALAT1 gene; if this gene is knocked out, this pathway will not be triggered and the possibility of tissue regeneration is reduced [28, 64]. In addition to the above-mentioned signaling pathways, ADSC-Exos can firstly accelerate the polarization of macrophages and next boost the secretion of pro-inflammatory factors by M2 macrophages [47, 65]. It has been shown in extensive studies that ADSC-Exos can translocate to macrophages, facilitate the polarization of macrophages, decrease the number of the inflammatory factors and elevate the mRNA expression of several anti-inflammatory related bio-factors, such as arginase-1 and interleukin-10 (IL-10), and finally inhibit the inflammatory response induced by lipopolysaccharide (LPS) and interferon (IFN-γ)stimulation of macrophages [45, 65, 66].

Of all the cytokines associated with the inflammatory response, interleukin-1β (IL-1β) and interleukin-6(IL-6) are paramount, being the key substances that influence the inflammatory response [41]. Macrophages are mainly divided into M1 macrophages, which are involved in the early inflammatory response and release various inflammatory factors, and M2 macrophages, which essentially stimulate the expression of anti-inflammatory factors and regulate stem cell differentiation [67, 68]. Among them, IL-1β is a pivotal cytokine that inhibits the polarization of macrophages, and it has the potential to stabilize the expression of additional inflammatory mediators [32]. In turn, IL-6 being an anti-inflammatory factor can guard against excessive inflammatory infiltrative responses and tissue damage [28]. Research has shown that ASC can ameliorate the inflammatory response by downregulating IL-1β while upregulating IL-6, thereby hastening tendon healing [28, 47].

A certain experiment revealed that the immunosuppressive function and the anti-inflammatory capacity of ASC-Exos are enhanced if the tendinopathy models are pretreated with inflammatory stimuli; thus, it is possible that the inflammatory stimuli underlie the release of immunotherapeutic exosomes from adipose tissue [69]. Nevertheless, because there is such a paucity of current experimental evidence that this result can also be achieved in tendon tissue, and since exosomes exert their immune properties through miRNA, it needs to be further investigated whether increasing stimulation of the inflammatory environment in a tendinopathy model can enhance the release of immune exosomes, while also looking at the role of miRNAs in them [69]. While the effect of miRNAs can be detected in partial experiments, specifically in reducing the inflammatory response, and while the expression of miR-let7 family is remarkable, further experiments are needed to verify whether these miRNAs play a role in the tendinopathy model [58]. Pre-treatment prior to experimentation refers not only to inflammatory stimuli, but also to hypoxia, chemicals, and cytokine modifications, all of which can directly or indirectly affect the function of stem cells and thus enhance the therapeutic potential of exosomes; once again, further experiments are needed to demonstrate that this plays the same role in tendon tissue [12].

### ASC-Exos stimulate the proliferation and migration of tenocytes

**Fig. 4:**
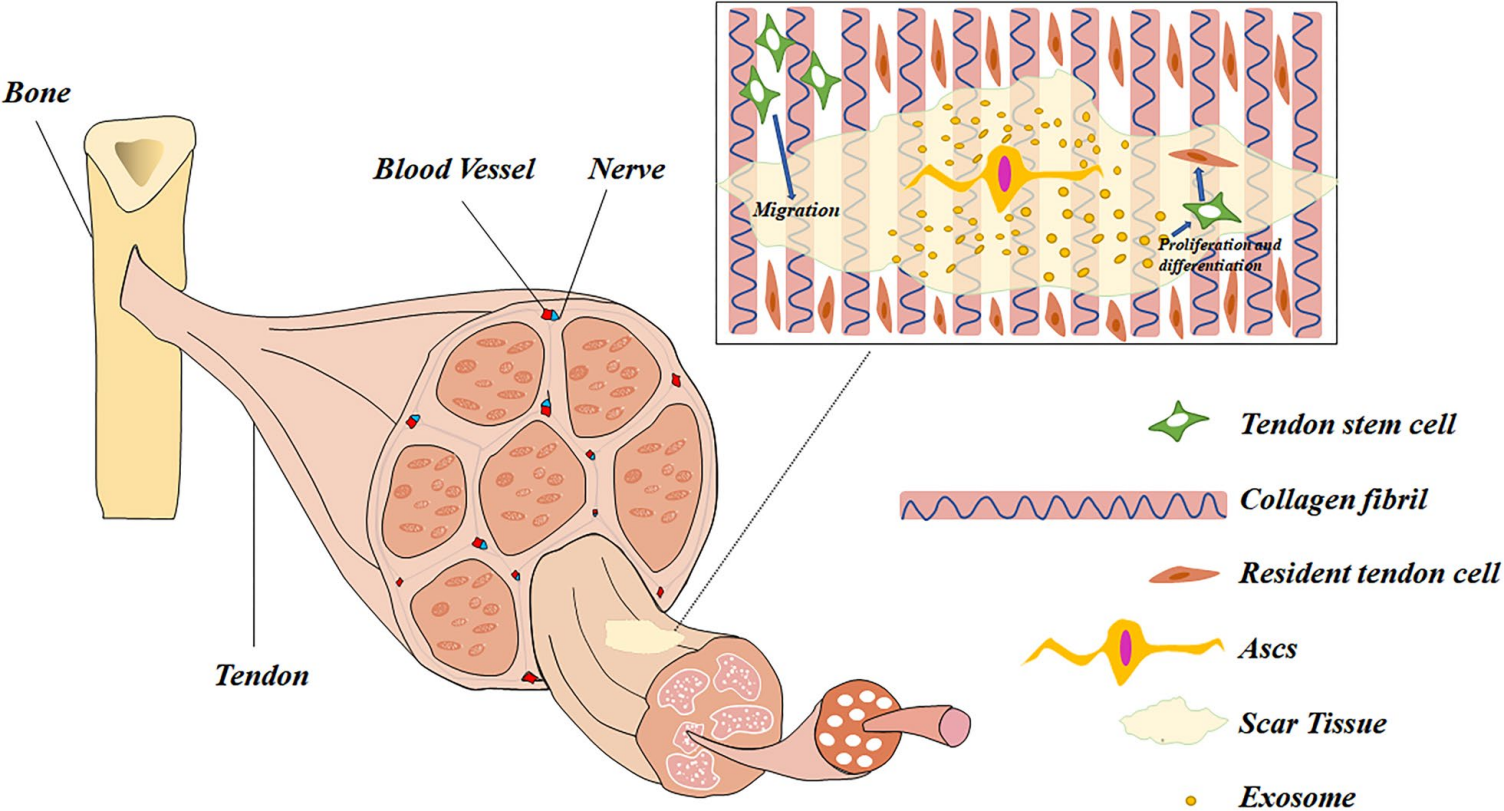
The process of ASC-Exos promoting tenocyte proliferation, differentiation and migration

In general, tendon differentiation is a sophisticated stage that involves multiple biomolecules interacting with one another, and the tendon cells account for the greatest proportion of variations in this process [70]. Some studies in the literature indicated that ADSC-Exos was able to significantly reduce apoptosis of tendon cells and myocytes and increase the migration and proliferation of tenocytes while augmenting the myogenesis of endogenous stem cells [18, 19, 59]. The process of ASC-Exos promoting tenocyte proliferation, differentiation and migration is shown in Fig. 4.

The most critical routes involved in tendon cell proliferation, migration, and differentiation are the MEK/ERK1/2 and PI3K/Akt and the SMAD2/3 and SMAD1/5/9 signaling pathway [19]. One of the most intimately linked molecules in the process of tendon cell proliferation and differentiation is SCXA, a basic helix–loop–helix transcription factor involved in the process of tendon regeneration and a specific marker for tendon progenitors and differentiated cells [42]. The process of tendon differentiation is closely correlated with the presence of SCXA, and it has also been shown to regulate the expression of genes of TNMD. TNMD is a transcription factor involved in tendon maturation and it also has a positive effect on the process of self-renewal of tendon cells [26, 71]. ASC-Exos activate the SMAD signaling pathway, thereby increasing the expression of TNMD and SCXA, and ultimately promoting tendon differentiation [26]. In principle, the migration rate of tendon cells peaks 12 h after treating tendon injuries with ADSC-Exos, which is attributed to the ability of ADSC-Exos to activate signaling pathways [19]. Related studies have revealed that there is a leucine-rich proteoglycan (biglycan) in the process of regulating tendon cell differentiation and migration, which is of great importance in the process of tendon healing [19]. ADSC-Exos can properly upregulate the amount of macroglycan, which will speed up the process of tendon healing. Not only this but the additional supplementation of desmoglein during the healing process will increase the mechanical properties of the tendon [19].

### ASC-Exos boost the synthesis of collagen

During the process of tendon healing, the fibrosis and scar formation of the tendon take place in a significant manner, and closely related to this procedure is the synthesis of dissimilar types of collagen [72]. The extracellular matrix is composed of three primary biomolecules: structural proteins, specialized proteins, and proteoglycans, with the heaviest proportion of type I collagen and a relatively minor proportion of additional collagen [1, 73]. Additionally, ECM remodeling is typically characterized by an increase in type III collagen accompanied by a decrease in type I collagen [28]. However, a significant number of investigations have demonstrated that persistent high expression of type III collagen will induce adhesions and scar formation in tendons, and the miR-29 family is closely associated with tendon fibrosis; therefore, it is necessary to pay close attention to the dosage and duration of ASC-Exos during treatment [28, 73, 74]. Specifically, ADSC-Exos promote the gene expression of collagen type I and the production of extracellular matrix, thereby promoting tendon regeneration [32].

The ratio of matrix metalloproteinase (MMPs) and its antagonist (metalloproteinase inhibitor) is related to the pathological changes of the tendon tissue, and the expression of MMPs/TIMPs is closely related to collagen synthesis [75, 76]. Among all MMPs, MMP-9 and MMP-13 can affect collagen synthesis to a large extent [28]. MMP-13 mostly degrades type 1 collagen, from which collagen protofibrils are broken down, laying a solid foundation for improving the mechanical properties of tendons [52, 77]. MMP-9, on the other hand, degrades smaller fragments similar to type III collagen [77]. ADSC-Exos significantly suppress the expression of MMP-9 and -13 genes, increasing the proportion of type I/III collagen genes, thereby promoting collagen synthesis and accelerating the process of tendon healing [28].

Other factors can impact collagen synthesis. For example, changes in the amount of type I collagen are mediated by the expression of tendon regulatory proteins, which correlate positively with the remodeling capacity of the ECM [19]. Additionally, tendon regulatory proteins can also adjust the proliferation and differentiation of tendon cells. It has been established that ADSC-Exos can indirectly induce the accumulation of type I collagen by significantly upregulating tendon regulatory proteins, thus expediting tendon healing [19]. It also has be demonstrated that ASC-Exos can upregulate tenascin, a tissue remodeling protein distributed in the abdomen of tendons, and they are closely associated with the number of collagen fibers and can regulate the direction of collagen fiber growth, making the maintenance of mechanical properties of tendons closely linked to this protein [55]. In all models of rotator cuff injury, tenascin is upregulated owing to its ability to maintain the mechanical properties of the tissue [55]. However, other models, such as the Achilles tendinopathy model and the patellar tendinopathy model, are required to validate this conclusion.

## Conclusions and future perspectives

Tendon injuries are a prevalent condition which are normally divided into two types: acute and chronic. Acute injury can progressively change to chronic injury if the acute injury is not promptly repaired [78, 79]. Tendon injury is mainly characterized by metabolic disorders of the cellular matrix, imbalance in the expression of matrix metalloproteinases and their inhibitors, as well as a remarkably long and slow treatment time requiring clinical observations of the efficacy of any treatment, therefore a sufficient number of trials are necessary to demonstrate the effectiveness of any therapeutic approach [75, 80].

At present, the methods used for tendon injury tend to be "cell-free therapy", and extracellular vesicles derived from mesenchymal stem cells have attracted much attention [49]. In particular, regarding the secretory group of stem cells, a large number of in vitro tests have been performed for exosomes. Due to the multiple strengths of adipose tissue, exosomes secreted by adipose-derived stem cells are more applicable to the treatment of the post-tendon injury. Extensive studies have established that ADSC-Exos can be effective in a wide range of diseases, especially cardiac, renal, neurological, and musculoskeletal disorders [30, 81]. The aspects of exosomes that play a role in distinct diseases include the following: accelerating skin wound healing, promoting immune system regulation, treating osteoarthritis, etc. [27].

The actions of ADSC-Exos in the healing process of tendon injuries are divided into four main parts: the effects of ADSC-Exos in the healing process of tendon injury are divided into four main components: facilitating angiogenesis, diminishing the inflammatory response, fostering the proliferation and migration of tendon cells, and speeding up collagen synthesis [26, 28, 30, 82].

Despite the roles and mechanisms of ASC-Exos in the treatment of tendon injuries described in this paper, some shortcomings remain. First, due to the excess of biological factors involved in the inflammatory response, in Table 4 we list only the most prominent of them, which are nearly all subject to regulation by ASC-Exos. Second, in describing the role and mechanism of ASC-Exos on tendon injury repair, which may contain uninvolved signaling pathways or various biomolecules, the paper only summarizes the results of the studies that have been argued to date; thus, more in-depth questions remain to be explored.

**Table 4:** Specific molecules contained in exosome

Molecules	Source	Biological effects
Biglycan	Adipose-derived stem cells	Biglycan is a leucine-rich repetitive proteoglycan, which is mainly important for the differentiation and migration of tendon cells, as well as for the regulation of collagen organization
Decorin	Adipose-derived stem cells	Decorin is a substance that significantly affects the mechanical properties of tendons
Prostaglandin-E2	Macrophages, adipose-derived stem cells	Prostaglandins-E2 are substances associated with inflammatory responses
Transforming growth factor-β	Adipose-derived stem cells, bone marrow mesenchymal stem cells, macrophages	Transforming growth factor-β can regulate T cells and participate in various immune responses, which also plays an essential role in the process of tendon fibrosis
Vascular endothelial growth factor	Adipose-derived stem cells, bone marrow mesenchymal stem cells, macrophages	Vascular endothelial growth factor plays an indispensable role in the process of tendon angiogenesis. Their main functions are to improve vascular permeability and enhance the proliferation of endothelial cells

Regarding problems related to the clinical application of exosomes, the following aspects need to be addressed: Firstly, the sample sizes used to date to conduct of ASC-Exos in vitro experiments remain too small and thus the conclusions drawn may be skewed; the majority of the experiments have been completed at the fourth week of analysis, which is not adequate time to check their validity. Therefore, in forthcoming studies, researchers need to use sufficient sample size and perform longer-term evaluations, which will provide ample evidence and accurate evaluation for the “cell-free therapy” [73, 83]. Secondly, the optimal concentration of exosomes for treatment has been established for models of wound healing, while the concentration for ASC-Exos to enhance tendon repair has not been validated by relevant trials [45, 80]. Thirdly, depending on the source of exosomes, the utilization of adipose tissue is considerably better than other types of tissues, which contain bone marrow, embryo, and amniotic membrane, but it is still worthwhile to conduct additional experiments to prove that exosomes can be extracted conveniently and are more efficient in treating tendon injuries [35]. A host of security issues regarding the extraction and preservation of exosomes still need to be addressed, especially to ensure their immunosuppressive capacity; standards must also be established for the storage stability of exosomes [22, 84].

Finally, acquiring more insights into the mechanism of action of ASC-Exos will help in defining their legal status and improving their therapeutic activity, which is crucial for their future clinical application. In conclusion, exosomes are deserving of further studies as a means of non-cellular therapy that reduces the potential pitfalls hidden by cellular therapy [19].

## Data Availability

Not applicable.

## Abbreviations

ASC-Exos: Adipose-derived stem cell of exosomes
(PI3K/Akt): Phosphatidylinositol-3-kinase/protein kinase B
Wnt: Wingless-associated integration site
TSCs: Tendon stem cells
HUMSCs: Human umbilical cord mesenchymal stem cells
BMSCs: Bone marrow mesenchymal stem cells
ASCs: Adipose-derived stem cells
VEGF: Vascular endothelial growth factorHIF-1α: hypoxia-inducible factors
MMP: Matrix metalloproteinase
FGF: Fibroblast growth factor
PDGF: Platelet-derived growth factors
AMPK: Adenosine 5ʹ-monophosphate (AMP)-activated protein kinase
LPS: Lipopolysaccharide
IFN-γ: Interferon
IL-1β: Interleukin-1β
IL-6: Interleukin-6
